# The Huntington's Disease-Related Cardiomyopathy Prevents a Hypertrophic Response in the R6/2 Mouse Model

**DOI:** 10.1371/journal.pone.0108961

**Published:** 2014-09-30

**Authors:** Michal Mielcarek, Marie K. Bondulich, Linda Inuabasi, Sophie A. Franklin, Thomas Muller, Gillian P. Bates

**Affiliations:** Department of Medical and Molecular Genetics, King's College London, London, United Kingdom; Emory University, United States of America

## Abstract

Huntington's disease (HD) is neurodegenerative disorder for which the mutation results in an extra-long tract of glutamines that causes the huntingtin protein to aggregate. It is characterized by neurological symptoms and brain pathology that is associated with nuclear and cytoplasmic aggregates and with transcriptional deregulation. Despite the fact that HD has been recognized principally as a neurological disease, there are multiple epidemiological studies showing that HD patients exhibit a high rate of cardiovascular events leading to heart failure. To unravel the mechanistic basis of cardiac dysfunction in HD, we employed a wide range of molecular techniques using the well-established genetic R6/2 mouse model that develop a considerable degree of the cardiac atrophy at end stage disease. We found that chronic treatment with isoproterenol, a potent beta-adrenoreceptor agonist, did not change the overall gross morphology of the HD murine hearts. However, there was a partial response to the beta-adrenergenic stimulation by the further re-expression of foetal genes. In addition we have profiled the expression level of *Hdacs* in the R6/2 murine hearts and found that the isoproterenol stimulation of *Hdac* expression was partially blocked. For the first time we established the *Hdac* transcriptional profile under hypertrophic conditions and found 10 out of 18 *Hdacs* to be markedly deregulated. Therefore, we conclude that R6/2 murine hearts are not able to respond to the chronic isoproterenol treatment to the same degree as wild type hearts and some of the hypertrophic signals are likely attenuated in the symptomatic HD animals.

## Introduction

Huntington's disease (HD) is an inherited neurodegenerative disorder caused by the expansion of a polyglutamine (polyQ) stretch within the huntingtin protein (HTT) [1]. The core features of HD are mainly neurological with a wide-spread brain pathology that is associated with the accumulation of toxic mutant huntingtin aggregate species [2]. In addition, HD is also characterised by peripheral pathological processes such as cardiac failure, weight loss and skeletal muscle atrophy [3], [4]. This might be explained by the ubiquitous expression of HTT and its fundamental biological functions in many cellular processes [2], [5], [6]. HTT is predicted to form an elongated superhelical solenoid structure due to a large number of HEAT motifs suggesting that it plays a scaffolding role for protein complex formation [6]. More than 200 HTT interaction partners have been identified which can be classified based on their function and include proteins that are involved in gene transcription, intracellular signalling, trafficking, endocytosis, and metabolism [7].

There are a number of factors to indicate that HD patients experience an HD-related heart pathology reviewed by Sassone et al [3]. This has been supported by multiple epidemiological studies that identified heart disease as the second cause of death in patients with HD [8]–[10]. A proof of concept study with an artificial transgenic mouse model expressing either a mutant polyQ peptide of 83 glutamines (PQ83) or a control peptide of 19 glutamines (PQ19), under the control of the α-myosin heavy chain promoter (MyHC) to drive cardiomyocyte-specific expression, showed a severe cardiac dysfunction and dilation leading to a reduced lifespan [11]. HD mouse models include the R6/2 and R6/1 lines, that are transgenic for a mutated N-terminal exon 1 HTT fragment [12] and the *Hdh*Q150 line in which an expanded CAG repeat has been knocked into the mouse *Htt* gene [13], [14]. Many pre-clinical studies have supported the hypothesis that mouse models of HD do indeed develop a cardiac dysfunction [15]–[18]. It has been demonstrated that R6/2 mice developed cardiac dysfunction by 8 weeks of age progressing to severe heart failure at 12 weeks with significant alterations in mitochondrial ultrastructure and increased levels of cardiac lysine acetylation [16]. In the HD symptomatic animals, pronounced functional changes have been previously showed by cardiac MRI revealing a contractile dysfunction, which might be a part of dilated cardiomyopathy (DCM). This was accompanied by the re-expression of foetal genes, apoptotic cardiomyocyte loss and a moderate degree of interstitial fibrosis but occurred in the absence of either mutant HTT aggregates in cardiac tissue or an HD-specific transcriptional deregulation.

R6/1 mice have also been shown to develop unstable R–R intervals that were reversed following atropine treatment, suggesting parasympathetic nervous activation, as well as brady- and tachyarrhythmias, including paroxysmal atrial fibrillation and sudden death. Collectively, R6/1 mice exhibited profound cardiac dysfunction related to autonomic nervous system that may be related to altered central autonomic pathways [17]. A recent study in the R6/2 and *Hdh*Q150 knock-in mouse models showed that the HD-related cardiomyopathy is caused by altered central autonomic pathways and was not due to the accumulation of toxic HTT aggregates as had previously been anticipated [11], [18].

In this study, we took advantage of the well-characterised beta-adrenergic agonist, isoproterenol. Chronic administration isoproterenol has been used in animals to study the mechanism of cardiac hypertrophy and failure [19]. Chronic infusion of isoproterenol has been reported to induce left ventricular hypertrophy accompanied by myocardial necrosis, apoptosis and fibrosis. Chronic isoproterenol infusion elicits alterations in cardiac gene expression that are consistent with the development of myocyte hypertrophy [20], [21]. Sympathetic nervous activation is a crucial compensatory mechanism in heart failure. However, excess catecholamine may induce cardiac dysfunction and beta-adrenergic desensitization [20], [21]. Altered alpha- and beta-adrenergic receptor signalling is associated with cardiac hypertrophy and failure. One of the main heart-related phenotypes in the R6/2 mouse model is a severe cardiac atrophy which may lead to cardiac failure [15]. Isoproterenol, a beta-adrenergic receptor (AR) agonist is known to induce myocardial hypertrophy and might prevent the HD-related cardiac phenotype [19]–[21]. We explored the importance of the HD axis in an isoproterenol-induced model of heart failure and tested the hypothesis that in pre-clinical settings, HD murine hearts might be resistant to isoproterenol action.

## Results

**Figure 1:**
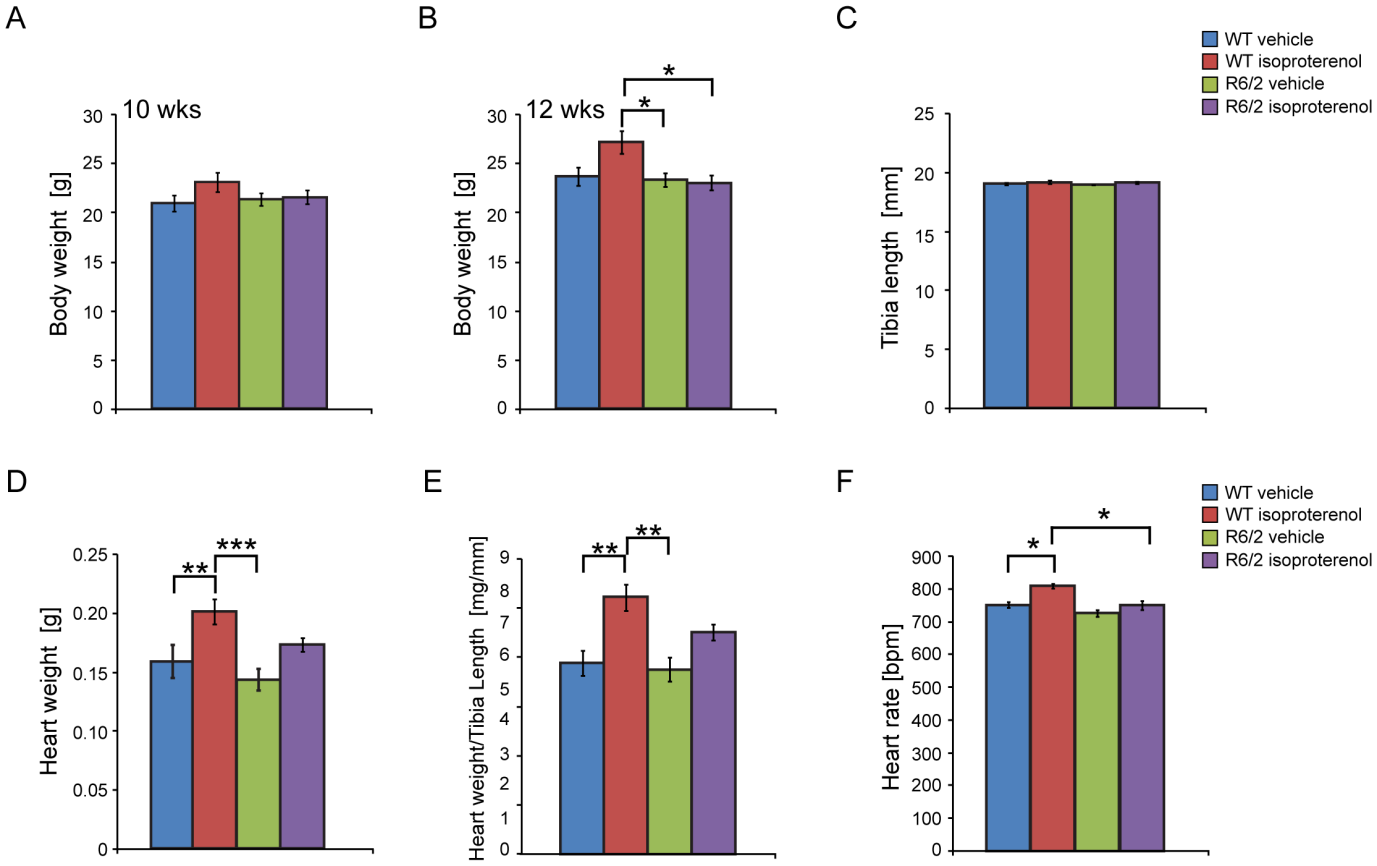
Morphometric analysis of the isoproterenol treated mice. (A) Body weight at 10 weeks of age prior to implantation of the alzet pumps. (B) Body weight (C) tibia length (D) heart weight (E) heart weight to tibia length index (F) heart rate were measured at 12 weeks of age. The gender-combined analysis of body weight did not detect a decrease in the R6/2 group, which may be because of a gender imbalance between the R6/2 and WT groups. All values are mean ± SEM (*n* = 8 WTveh, *n* = 9 WTiso, *n* = 14 R6/2veh, *n* = 14 R6/2iso). One-way ANOVA with Bonferroni *post-hoc* test: *p<0.05, **p<0.01, ***p<0.001.

**Figure 2:**
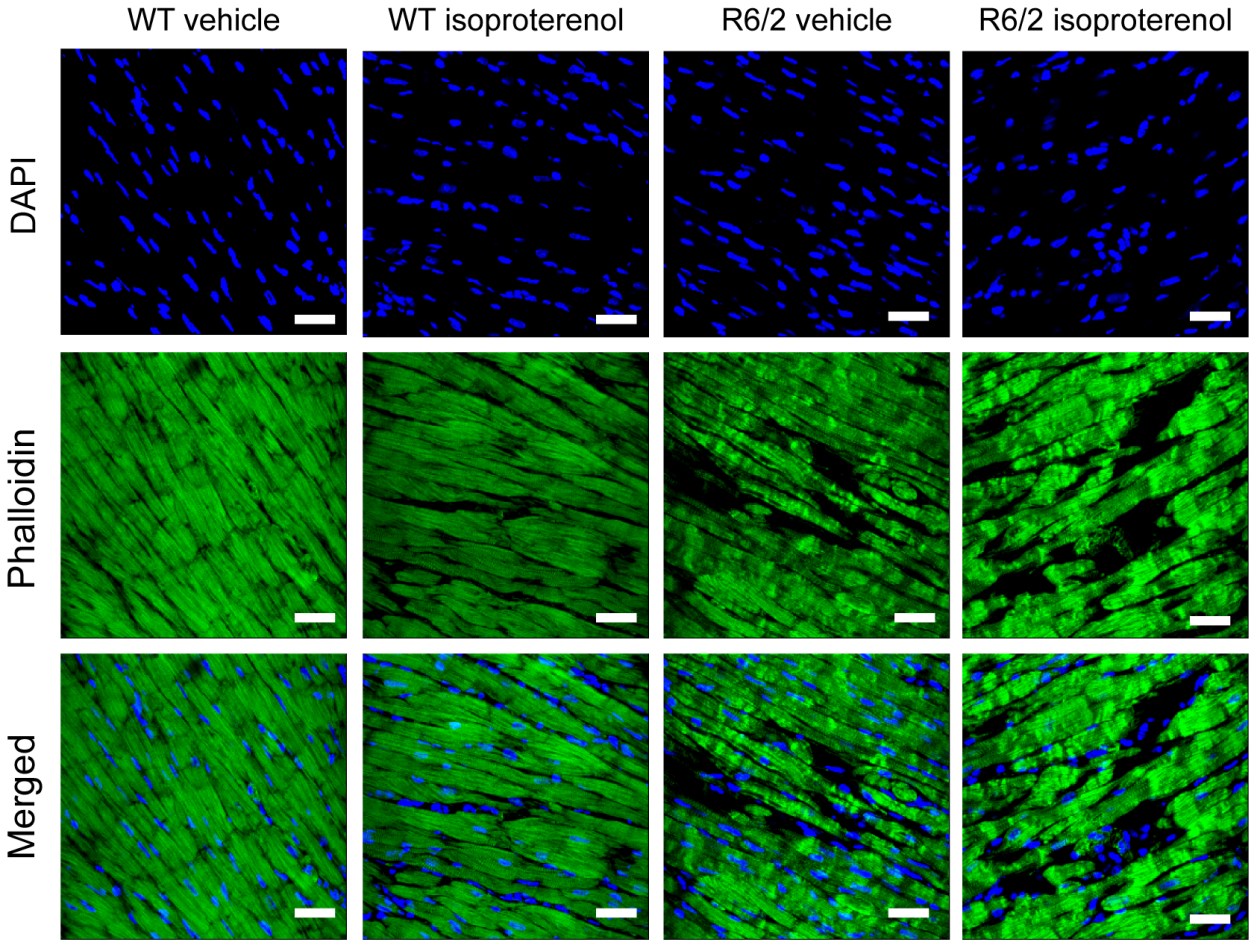
Gross cardiac morphology of the hearts treated with isoproterenol. Representative phalloidin staining (green) shows left ventricle myocyte hypertrophy in WT mice but not in 12 week old R6/2 mice. Nuclei (blue) were visualized with DAPI. Scale bar 30 µm.

We tested the hypothesis that induction of myocardial hypertrophy might be prevented by the HD-related cardiac phenotypes. We administered isoproterenol (Iso) to symptomatic R6/2 mice from 10 weeks of age for two weeks. The wild type (WT) and R6/2 mice had comparable body weights at both the start (Figure 1A) and end of the trial (Figure 1B) and comparable tibia length at 12 weeks of age (Figure 1C). As expected, the heart weight was significantly increased in the WT (Iso) group but surprisingly not in the R6/2 (Iso) mice (Figure 1D). Consequently, the HW/TL index (heart weight to tibia Length) was significantly increased in the WT (Iso) group in comparison to the WT vehicle (veh) group but there was no significant change between R6/2 (Iso) and R6/2 (veh) groups (Figure 1E). Next, to determine whether isoproterenol treatment triggered an increased in heart rate, we examined electrocardiogram (ECG) recordings in conscious mice at the end of trial. We found that, WT (Iso) but not R6/2 (Iso) treated mice had a significantly higher heart rate in comparison to their respective vehicle groups (Figure 1F). We conclude from this morphometric analysis that R6/2 mice do not respond to chronic isoproterenol treatment. This was supported by the visualisation of cardiomyocyte gross morphology with phalloidin staining as shown in Figure 2. We have previously described the cardiomyocyte loss in the hearts of R6/2 mice [15] and the phalloidin staining indicated that isoptroterenol treatment of R6/2 mice does not lead to an improvement in cardiomyocyte disarray or to an increase in cardiomyocytes size (Figure 2).

**Figure 3:**
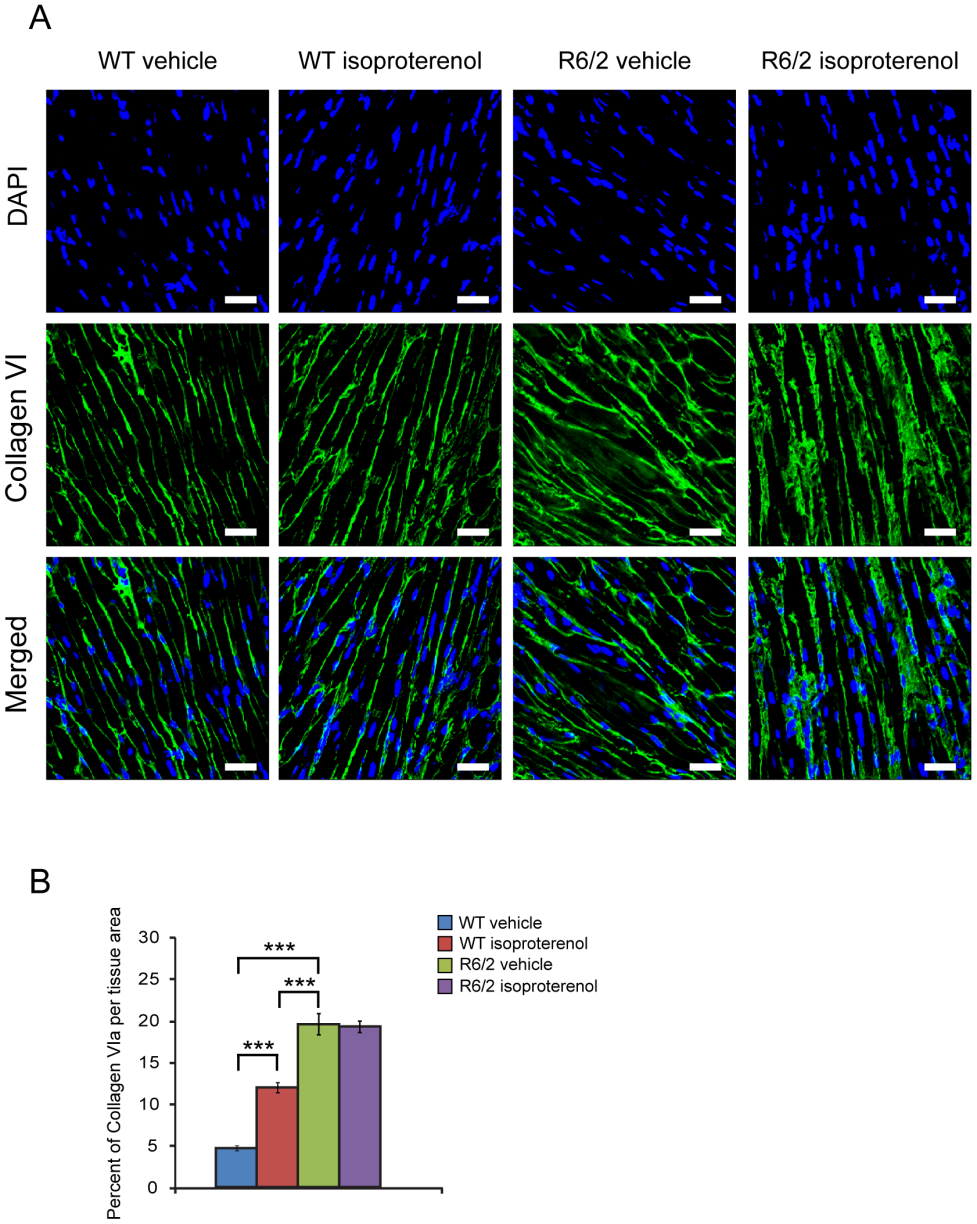
Moderate fibrosis level based on collagen VI deposits is not attenuated in the hearts of R6/2 mice. (A) Representative confocal pictograms of whole heart sections from 12 week old WT and R6/2 mice. (B) Quantification of the collagen VI staining area. Fibrosis was detected with the anti-collagen VI antibody (green) and nuclei (blue) were visualised with DAPI. Scale bar 30 µm. Values are mean ± SEM (*n* = 3). Student's *t* test: *p<0.05, ***p<0.001.

It is well established that chronic treatment with isoproterenol may lead to increased fibrosis. As we previously reported, R6/2 mice develop an interstitial type of fibrosis [15] and this has can be visualised in (Figure 3A). As expected WT (iso) mice displayed a higher degree of fibrosis than their vehicle controls, while R6/2 (Iso) mice did not develop further fibrotic deposits in comparison to the R6/2 vehicle group (Figure 3B).

**Figure 4:**
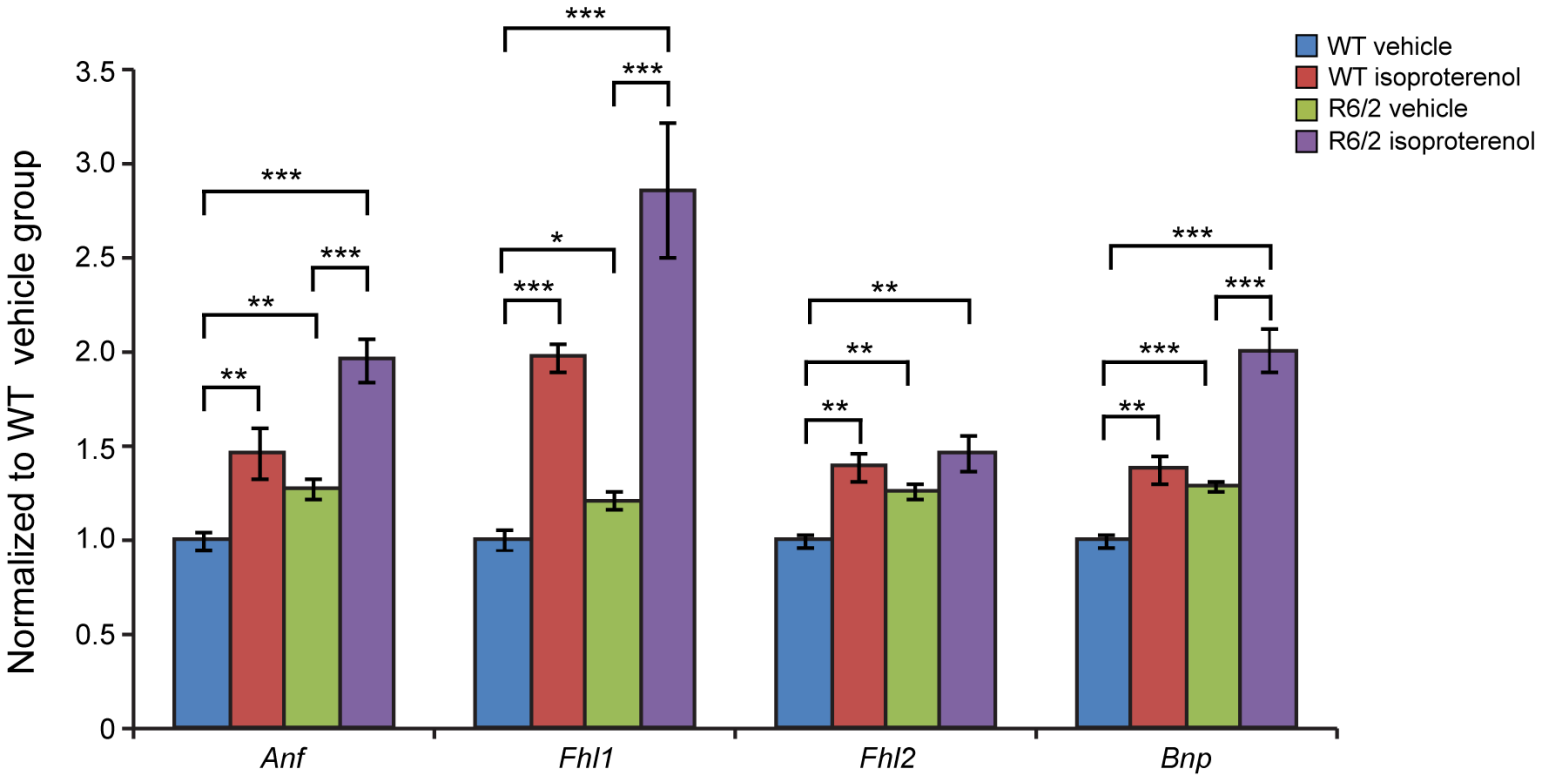
Partial re-activation of foetal gene markers in the hearts of R6/2 treated with isoproterenol. *Anp* (atrial natriuretic peptide), *Bnp* (brain natriuretic protein) and members of the four and half LIM family *Fhl1* and *Fhl2 were* elevated in the heart of WT and R6/2 mice. All Taqman qPCR values were normalized to the geometric mean of three housekeeping genes: *Actb*, *Cyc1* and *Gapdh*. Error bars are SEM (n = 6). Two-way ANOVA with Bonferroni *post-hoc* test: *p<0.05, **p<0.01; ***p<0.001.

**Figure 5:**
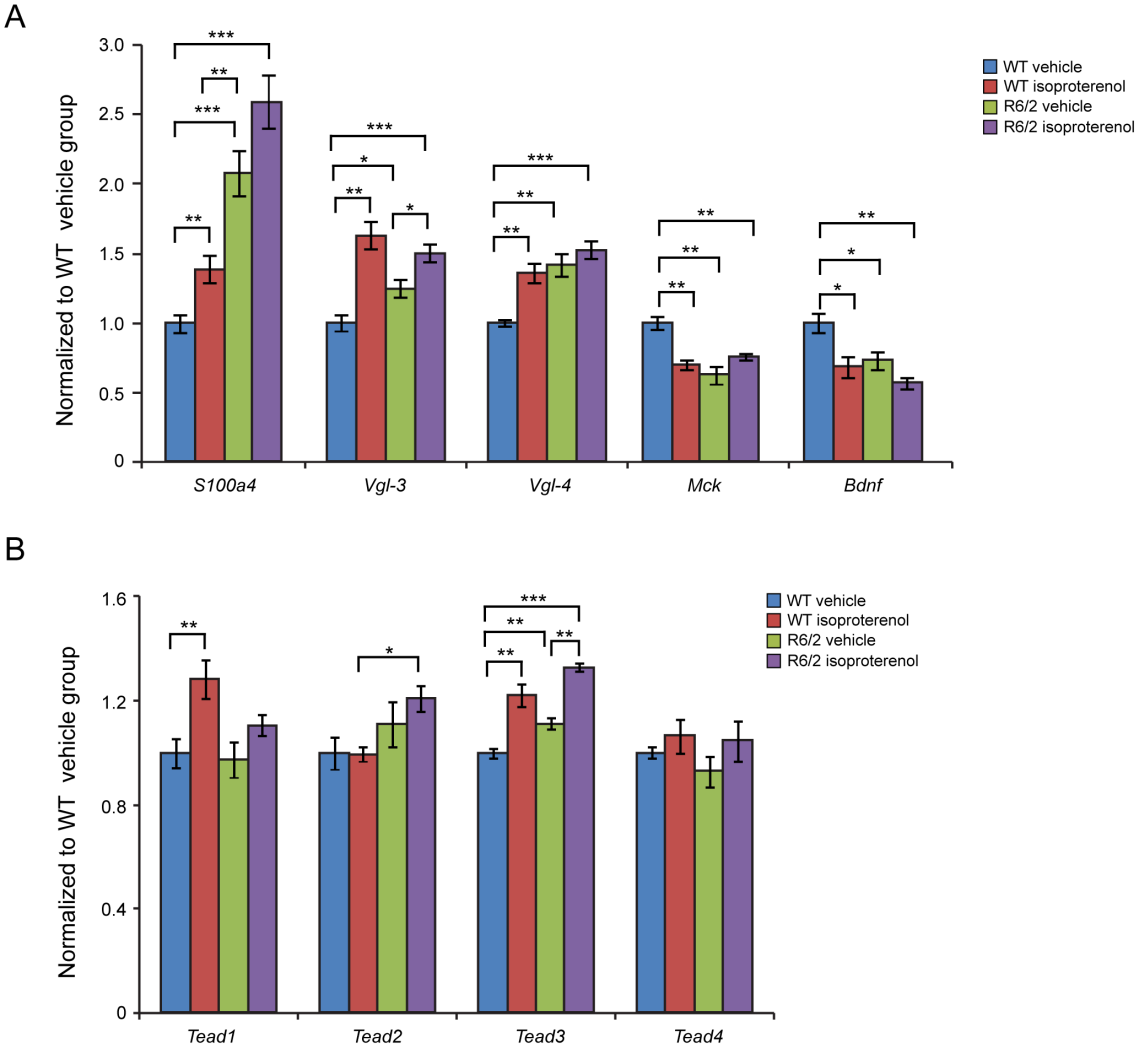
Transcriptional deregulation of HD markers involved in heart failure. (A) *S100A4* (S100 calcium binding protein A4), Vgl-3 (vestigial related factor 3), *Vgl-4* (vestigial related factor 4) were up-regulated while *Mck* (muscle creatine kinase) and *Bdnf* (brain derived neurotophic factor) transcripts were significantly decreased in the heart of R6/2 mice. (B) Transcripts of Transcriptional Enhancer Family members (TEAD or TEFs) were significantly deregulated in isoproterenol treated mice. All Taqman qPCR values were normalized to the geometric mean of three housekeeping genes: *Actb*, *Cyc1* and *Gapdh*. Error bars are SEM (n = 6). Two-way ANOVA with Bonferroni *post-hoc* test: *p<0.05, **p<0.01; ***p<0.001.

Pathological changes in the heart are often associated with the reactivation of a foetal gene programme and therefore, we assessed the expression levels of genes known to be changed as a consequence of cardiac hypertrophy or dilated cardiomyopathy (DCM). We found *Anp* (atrial natriuretic peptide) and *Bnp* (brain natriuretic peptide) to be up-regulated in WT (Iso) mice as well in the R6/2 (Iso) animals in comparison to their respective vehicle groups (Figure 4). Two members of the four and half only LIM family, namely *Fhl1* and *Fhl2*, are also typically reactivated foetally-expressed genes. Both transcripts showed a significant up-regulation in the R6/2 and WT isoproterenol treated mice (Figure 4). To further examine the degree of heart pathology, we determined the expression levels of additional genes that are typically altered in the HD diseased hearts. The multifunctional Ca^2+^-binding protein *S100A4* has been shown to be up regulated in the R6/2 mice [15]. However, isoproterenol treatment did not cause a further up-regulation of *S100A4* transcripts while a 50% fold induction has been observed in WT (Iso) animals (Figure 5A). *Vgl-4* (vestigial related factor 4) and *Vgl-3* (vestigial related factor 3) are vital co-activators of the TEF (transcription enhancer family) and have been anticipated to be markers of cardiac hypertrophy [22]–[25]. *Vgl-3* mRNA was significantly up-regulated in both WT and R6/2 isoproterenol treated mice and in R6/2 mice in comparison to WT littermates (Figure 5A), while *Vgl-4* transcripts were only elevated in the WT (Iso) group but not in R6/2 (Figure 5A). As previously shown *Vgl-4* transcripts were up-regulated in the R6/2 mice in comparison to WT animals (Figure 5A) [15].

*Bdnf* (brain derived neutrophic factor) is down-regulated in the brain of HD mouse models [26] and we previously found that its transcripts were decreased in the hearts of R6/2 mice [15]. Isoproterenol treatment caused a significant reduction of *Bdnf* mRNA in WT animals but no further reduction has been observed in the R6/2 (Iso) group (Figure 5A). A similar profile of transcriptional deregulation was observed for *Mck* (muscle creatinine kinase) (Figure 5A). Transcriptional enhancer family (TEFs) members' (TEAD) have been described to be involved in the development of cardiac hypertrophy in rodents [27]–[29]. In the isoproterenol treated WT animals a significant induction of their transcripts was detected for *Tead-1* and *Tead-3* (Figure 5B). None of four TEF members was found to be deregulated in the symptomatic R6/2 mice (Figure 5B). Only *Tead-3* showed a significant further up-regulation upon isoproterenol treatment in the hearts of R6/2 animals (Figure 5B).

The heart responds to pathological stresses by remodelling in a manner that is associated with myocyte hypertrophy and recent studies suggest key roles for histone deacetylases (HDACs) in the control of pathological cardiac remodelling [30]–[32]. Hence, first we sought to monitor the transcriptional profile of *Hdacs* and *Sirtuins* in the R6/2 mouse model. We performed a longitudinal study in the R6/2 mouse model from 4 weeks of age (presymptomatic) to 15 weeks (symptomatic) to evaluate the transcriptional changes of 11 *Hdacs* (Figure S1) and 7 *Sirtuins* (Figure S2). *Hdac 3* and *Hdac9* were dysregulated in 4 week old R6/2 hearts (Figure S1A), but by 8 weeks of age the expression level of all *Hdacs* was comparable to WT. (Figure S1B). However, in the symptomatic R6/2 animals at 12 and 15 weeks of age, we found a significant up-regulation of *Hdac1*, *Hdac4*, *Hdac5*, *Hdac6* and *Hdac8* while *Hdac3* mRNA was markedly decreased (Figure S1C and D). Of the 7 *Sirtuins*, *Sirt 6* and *Sirt 7* transcripts were upregulated at 8 weeks of age, *Sirt 1* at 12 weeks and *Sirt4* and *Sirt6* at the end stage of disease in R6/2 mouse models hearts (Figure S2).

**Figure 6:**
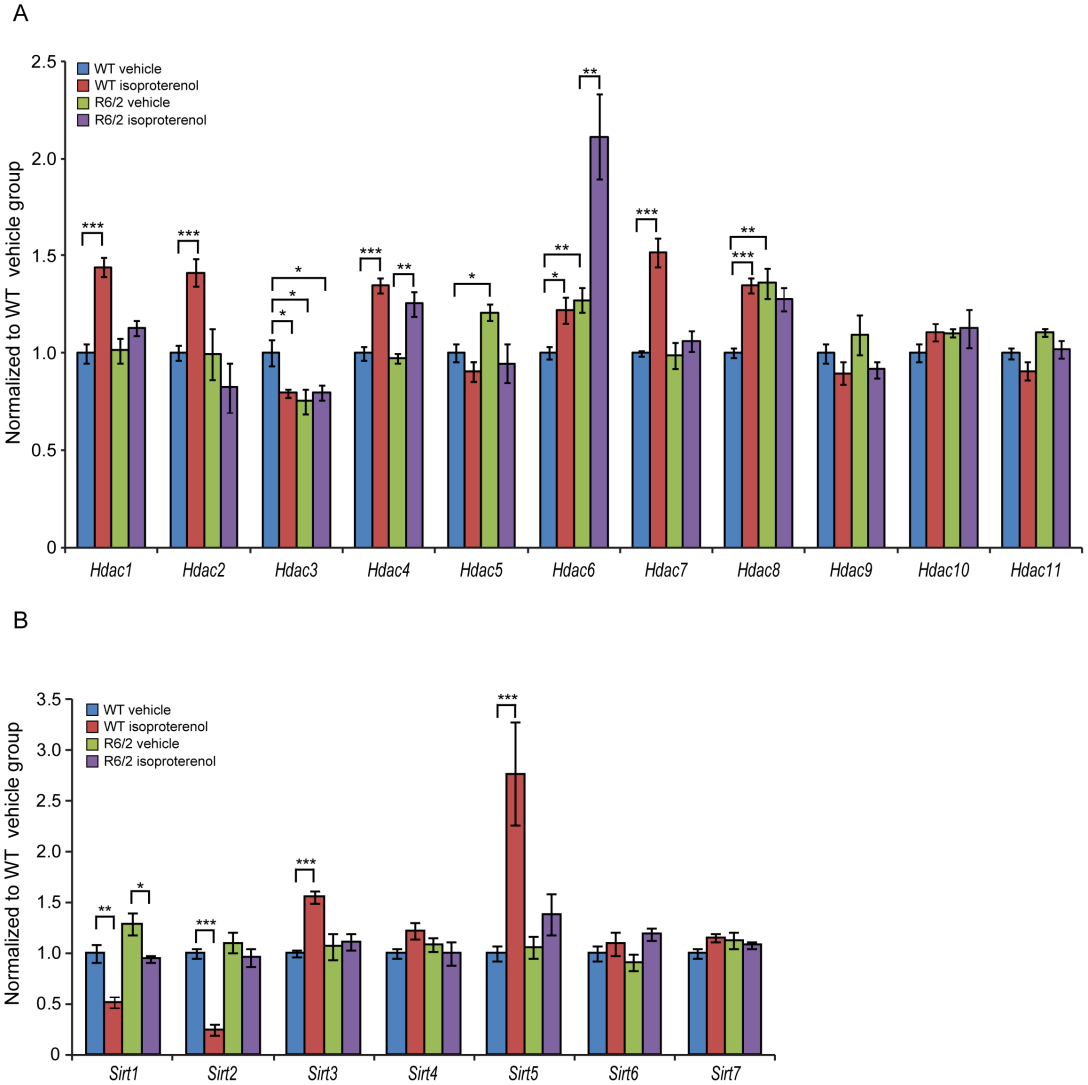
Chronic administration of isoproterenol causes a significant transcriptional deregulation of many *Hdacs* and *Sirtuins*. (A) *Hdac1*, *Hdac2*, *Hdac4*, *Hdac6*, *Hdac7* and *Hdac8* transcript levels were increased while *Hdac3* mRNA was significantly reduced in the heart of WT mice treated with Isoproterenol. Only *Hdac4* and *Hdac6* were significantly increased in the hearts of R6/2 mice. (B) *Sirt3* and *Sirt5* transcript levels were significantly increased in the heart of WT mice treated with Isoproterenol. *Sirt 1* and *Sirt 2* were decreased in the hearts of WT mice, but only *Sirt2* was decreased in the hearts of R6/2 mice. All Taqman qPCR values were normalized to the geometric mean of three housekeeping genes: *Actb*, *Cyc1* and *Gapdh*. Error bars are SEM (n = 6). Two-way ANOVA with Bonferroni *post-hoc* test: *p<0.05, **p<0.01; ***p<0.001.

For the first time, we showed that chronic isoproterenol treatment caused up-regulation of *Hdac1*, *Hdac2*, *Hdac4*, *Hdac6*, *Hdac7* and *Hdac8* transcripts and down-regulation of *Hdac3* mRNA in WT mice (Figure 6A). In the R6/2 isoproterenol treated mice, we only found a significant deregulation of *Hdac4* and *Hdac6* (Figure 6A). *Sirtuins* showed a minor degree of transcriptional changes with a significant down-regulation of *Sirt1* and *Sirt2*, while transcripts of *Sirt3* and *Sirt5* were significantly up-regulated in the WT animals (Figure 6B). Only *Sirt1* mRNA was significantly down-regulated in the hearts of R6/2 isoproterenol treated mice (Figure 6B). Overall, one might conclude that chronic stimulation of beta-adrenergic receptors in the hearts of WT mice led to a significant deregulation of specific *Hdacs* and *Sirtuins*.

## Discussion

Cardiac hypertrophy represents a critical compensatory mechanism to hemodynamic stress or injury. HD-related cardiomyopathy has been recently characterised by aberrant gap junction channel expression and a significant deregulation of hypertrophic markers that may predispose them to arrhythmia and an overall change in cardiac function. These changes were accompanied by the re-expression of foetal genes, apoptotic cardiomyocyte loss and a moderate degree of interstitial fibrosis in the symptomatic animals [15].

It has been previously reported that the heart rate response to the maximal single dose of isoproterenol was attenuated in the symptomatic R6/1 HD mouse model but not in pre-symptomatic animals in comparison to the WT littermates [17] and this might indicate that the hypertrophic response in HD hearts is attenuated. One of the strategies to unravel the mechanism of cardiac hypertrophy and failure is a chronic administration of the beta-adrenergic receptor agonist isoproterenol [19]. Chronic infusion of Iso has been reported to induce left ventricular systolic and diastolic dysfunction and left ventricular hypertrophy accompanied by myocardial apoptosis and necrosis [20], [21]. As heart atrophy and hypertrophy are governed by similar pathways, a better understanding of the cross talk between them may also contribute to an elucidation of the mechanism of HD-related cardiomyopathy. Since the proliferative capacity of adult cardiomyocytes is rather limited, the regulation of heart size is based on hypertrophy and atrophy at the cellular level. Interestingly the beta-adrenergic receptor densities were not altered in the R6/2 animals based on immunohistochemistry as has been shown previously [16].

In this study we aimed to provide a broad spectrum of experimental insights into the hypertrophic response in the hearts of R6/2 mouse model of HD. A cardiac morphometry revealed that HD hearts were not responsive to hypertrophic stimuli in the symptomatic animals while WT animals had developed all of the typical characteristics of hypertrophic hearts including increased heart weight, HW/TL index and increased heart rate in comparison to vehicle groups. Similarly, based on immunohistochemistry, we did not find an increased fibrosis in the hearts of R6/2 (Iso) mice while WT animals developed a significantly higher level of fibrotic deposits. In addition, cardiac gross morphology did not change upon isoproterenol treatment and there was no obvious cardiomyocyte hypertrophy, although the pronounced cardiomyocyte disarray was still observed in the Iso treated hearts of R6/2 mice. In contrast to the morphometry and immunohistochemistry analysis, we found further re-activation of foetal genes such as *Anf*, *Fhl1* and *Bnp* but not *Fhl2* in the isoproterenol treated R6/2 hearts. This might suggest that the HD hearts were able to respond at least partially to beta-adrenergic stimulation. It has to be noted that the WT animals responded as expected and the levels of all examined transcripts were significantly increased.

In addition, we analysed the expression pattern of transcripts known to be deregulated in HD mouse models [15]. We noted that chronic administration of Isoproterenol did not further modify expression of *S100A4*, *Vgl-4*, *Vgl-3*, *Mck* and *Bdnf*. In addition, we found for the first time that beta adrenergic stimulation was sufficient to modulate the expression levels of *S100a4*, *Vgl-3*, *Vgl-4* in WT animals, underlining their function in heart hypertrophy. It has been previously proposed that TEA domain transcription factor-1 (TEAD-1) is essential for proper heart development. It is implicated in cardiac specific gene expression and the hypertrophic response of primary cardiomyocytes to hormonal and mechanical stimuli, and its activity increases in the pressure-overloaded hypertrophied hearts. Hence, TEAD family members have been proposed to play a crucial role in the hypertrophic response [28]. For the first time we identified that *Tead-1* and *Tead-3* are the major players in the hypertrophic response as their transcripts levels were significantly increased in WT animals treated with isoproterenol. In the R6/2 mice there was no further up-regulation of all 4 isoforms of the TEAD family. However, only *Tead-3* was clearly significantly up-regulated in the R6/2 mice treated with isoproterenol. Also the expression pattern of *Mck* and *Bdnf* transcripts in R6/2 hearts was similar to that in WT animals treated with isoproterenol and was not further modified in the R6/2 Iso treated mice.

Global protein acetylation was found to be increased in the symptomatic R6/2 hearts based on immunohistochemistry [16] and it is well know that epigenetic remodelling is crucial for cellular differentiation and development. HDACs in the heart control events such as hypertrophy [33], [34], fibrosis [35], contractility [36] and energy metabolism [37]. Global HDAC activity is increased in the hypertrophic rat hearts [38] and in a model of cardiac ischemia-reperfusion injury [39]. The mechanisms by which HDAC inhibitors suppress pathological cardiac hypertrophy are still being elucidated. Previous studies has shown that HDAC inhibitors can reduce cardiac hypertrophy under pathological conditions [34], [38] and may also attenuate structural remodelling after myocardial infarction [40]. Hence, we were interested in profiling the expression pattern of all 18 HDACs in the R6/2 hearts. We found that *Hdac1*, *Hdac3*, *Hdac4*, *Hdac5*, *Hdac6* and *Hdac8* were significantly deregulated in the murine R6/2 hearts at the end stage of disease. Our longitudal analysis clearly showed that there was no difference in the expression of all *Hdacs* in early symptomatic animals. Similarly we found that *Sirt4* and *Sirt6* transcripts were significantly up-regulated in the fully symptomatic animals.

In addition, we found the following *Hdacs* to be significantly deregulated in the isoproterenol treated murine WT hearts: *Hdac1*, *Hdac2*, *Hdac3*, *Hdac4*, *Hdac6*, *Hdac7*, *Hdac8*, *Sirt1*, *Sirt2*, *Sirt3* and *Sirt5*. Some of the findings are in line with a previous study and confirmed a role for *Hdacs* in hypertrophic signalling. For example, it has been shown that *Hdac2* over-expression provokes severe cardiac hypertrophy [41]. *Hdac3* over-expression in the heart resulted in cardiomyocyte hyperplasia [41]. Silencing of *Hdac5* [42] and *Hdac9* [43] resulted in an exaggerated hypertrophic response to the pressure overload and spontaneous hypertrophy in older animals while *Sirt1* inhibition resulted in enhanced apoptosis [44]. In the R6/2 mouse model, chronic isoproterenol treatment caused a further deregulation of *Hdac4*, *Hdac6* and *Sirt1* while the expression profile of others *Hdacs* remained unchanged. This might indicate that chronic treatment with isoproterenol was able to partially replicate the expression pattern observed in the WT animals and it is likely that some hypertrophic pathways are altered in the HD mouse models. In conclusion, our present work shed light on mutant HTT as novel modulator of cardiac function. Given that beta-adrenergic signalling is a vital regulator of myocardial function, it will be important to elucidate and explore new pathways to further understand this complex cardiac response in HD mouse models and potentially in clinical settings.

## Materials and Methods

### Ethics statement

All experimental procedures performed on mice were conducted under a project licence from the Home Office and approved by the King's College London Ethical Review Process Committee.

### Mouse maintenance and breeding

Hemizygous R6/2 mice were bred by backcrossing R6/2 males to (CBA x C57BL/6) F1 females (B6CBAF1/OlaHsd, Harlan Olac, Bicester, UK). All animals had unlimited access to water and breeding chow (Special Diet Services, Witham, UK), and housing conditions and environmental enrichment were as previously described [45]. Mice were subject to a 12-h light/dark cycle. All experimental procedures were performed according to Home Office regulations.

### Genotyping

Genomic DNA was isolated from an ear-punch. R6/2 mice were genotyped by PCR and the CAG repeat length was measured as previously described [46] and listed in Table S1. Dissected tissues were snap frozen in liquid nitrogen or embedded in OCT and stored at −80°C until further analysis.

### Chronic treatment with Isoproterenol

Isoproterenol hydrochloride was prepared fresh, diluted in PBS (SIGMA, I6504-1G lot 018K5003). Mini osmotic pumps (Alzet pumps Model 2002, Charles River 0000296) were loaded with 200 µl of either vehicle or isoproterenol at a dose of 220 µg/g/day to allow diffusion at 0.5 µl/hour for 14 days. Animals were initially anesthetized with 5% isoflurane, and then anaesthesia was maintained at ∼1.5% isoflurane throughout the surgical procedure. Alzet pumps were implanted subcutaneously onto the back of the mouse and the skin was stapled together using wound clippers. After 14 days the mice were culled and their hearts taken for analysis. Mice were randomised from litters born as closely matched as possible. Body weight was measured at the beginning and the end of the trial.

### RNA extraction and Taqman real-time PCR expression analysis

Total RNA from whole hearts was extracted with the mini-RNA kit according to manufacturer's instructions (Qiagen). The reverse transcription reaction (RT) was performed using MMLV superscript reverse transcriptase (Invitrogen) and random hexamers (Operon) as described elsewhere [47]. The final RT reaction was diluted 10-fold in nuclease free water (Sigma). All Taqman qPCR reactions were performed as described previously [48] using the Chromo4 Real-Time PCR Detector (BioRad). Estimation of mRNA copy number was determined in triplicate for each RNA sample by comparison to the geometric mean of three endogenous housekeeping genes (Primer Design) as described [49]. Primer and probe sets for genes of interest were purchased from Primer Design or ABI.

### Immunohistochemistry and confocal microscopy

For immunohistochemical studies, hearts were snap frozen in liquid nitrogen, or frozen in isopentane at −50°C, or incubated overnight in 4% PFA followed by overnight incubations in 20% and 30% sucrose in PBS, prior to embedding in OCT and storage at −80°C. 10–15 µm sections were cut using a cryostat (Bright instruments), air dried and immersed in 4% PFA in PBS or in acetone at −20°C for 15 min and washed for 3×5 min in 0.1% PBS-Triton X-100. Blocking was achieved by incubation with 5% BSA-C (Aurion) in 0.1% PBS-Triton X-100 for at least 30 min at RT. Immunolabeling with primary antibodies was performed in 0.1% PBS-Triton X-100, 1% BSA-C overnight in a humidity box at 4°C as described previously [15]. Sections were washed 3× in PBS, incubated for 60 min at RT in a dark box with the anti-rabbit (FITC Invitrogen 1∶1000 in PBS), washed 3× in PBS and counterstained with DAPI (Invitrogen). Sections were mounted in Vectashield mounting medium (Vector Laboratories). Sections were examined using the Leica TCS SP4 laser scanning confocal microscope and analysed with Leica Application Suite (LAS) v5 (Leica Microsystems, Heidelberg, Germany).

### ECG evaluation in conscious mice

Heart rate was monitored using the ECGenie apparatus (Mouse Specifics, Inc., Boston, MA, USA). This device is a PowerLab-based system that acquires signal through disposable footpad electrodes located in the floor of a 6.5 cm by 7 cm recording platform. Heart rate was determined from the average of the RR interval.

### Statistical analysis

All data were analysed with Microsoft Office Excel and Student's *t*-test (two tailed) or TWO-WAY ANOVA with Bonferroni *post-hoc* test SPSS (IBM).

## Supporting Information

Figure S1 **Longitude changes in** ***Hdac*** **gene expression in the hearts of R6/2 mice.** Transcript levels of 11 *Hdacs* were monitored in the hearts of pre- and symptomatic R6/2 mice at (A) 4 weeks, (B) 8 weeks, (C) 12 weeks and (D) 15 week of age. All Taqman qPCR values were normalized to the geometric mean of three housekeeping genes: *Actb*, *Cyc1* and *Gapdh*. Error bars are SEM (n = 6). Student *t-test*: *p<0.05, **p<0.01; ***p<0.001.
(TIF)

Figure S2 **Longitude changes in the** ***Sirtuin*** **expression in the hearts of R6/2 mice.** Transcript levels of 7 *Sirtuins* were monitored in the hearts of pre- and symptomatic R6/2 mice at (A) 4 weeks, (B) 8 weeks, (C) 12 weeks and (D) 15 weeks od age. All Taqman qPCR values were normalized to the geometric mean of three housekeeping genes: *Actb*, *Cyc1* and *Gapdh*. Error bars are SEM (n = 6). Student *t-test*: *p<0.05, **p<0.01; ***p<0.001.
(TIF)

Table S1 **Summary of the number of mice per genotype used in all studies and their CAG repeat sizes.** SD = standard deviation.
(DOC)
