# Supplementary figures and images for: The Huntington's Disease-Related Cardiomyopathy Prevents a Hypertrophic Response in the R6/2 Mouse Model
Source: PLoS One. 2014 Sep 30;9(9):e108961. doi: 10.1371/journal.pone.0108961 (PMC4182603; doi:10.1371/journal.pone.0108961)

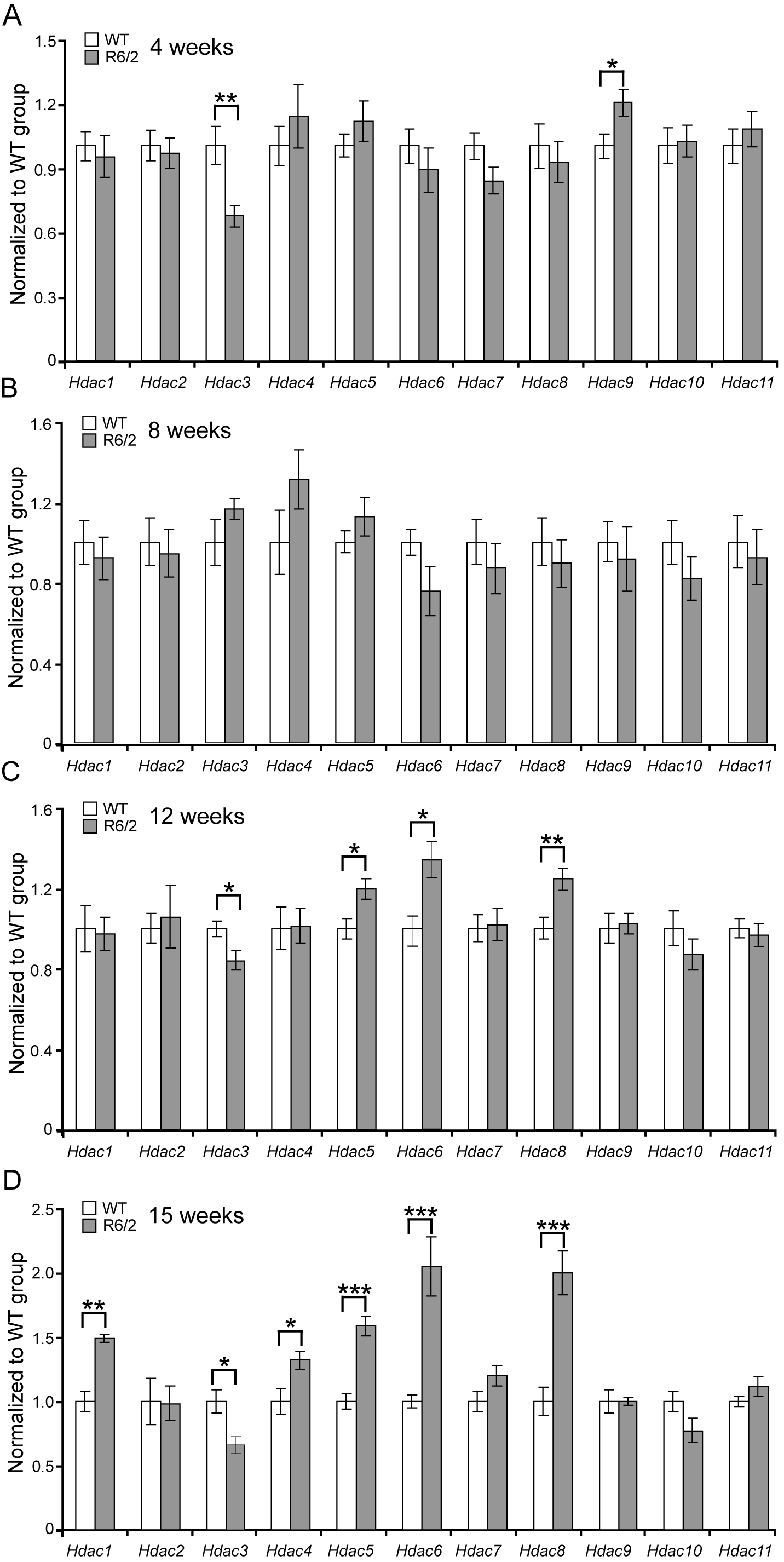

Supplement: Figure S1 — Longitude changes in Hdac gene expression in the hearts of R6/2 mice. Transcript levels of 11 Hdacs were monitored in the hearts of pre- and symptomatic R6/2 mice at (A) 4 weeks, (B) 8 weeks, (C) 12 weeks and (D) 15 week of age. All Taqman qPCR values were normalized to the geometric mean of three housekeeping genes: Actb, Cyc1 and Gapdh. Error bars are SEM (n = 6). Student t-test: *p<0.05, **p<0.01; ***p<0.001. (TIF) [file pone.0108961.s001.tif]

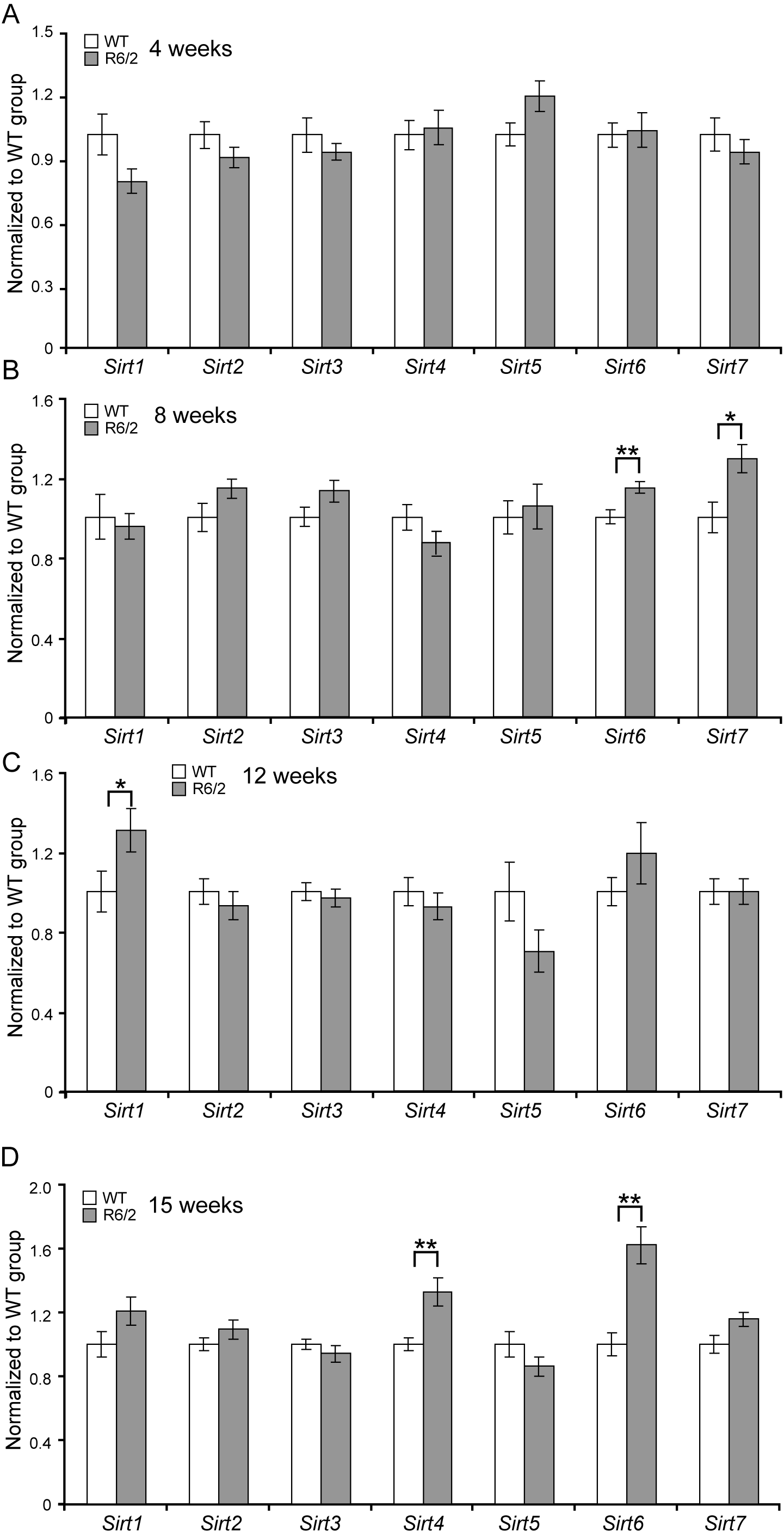

Supplement: Figure S2 — Longitude changes in the Sirtuin expression in the hearts of R6/2 mice. Transcript levels of 7 Sirtuins were monitored in the hearts of pre- and symptomatic R6/2 mice at (A) 4 weeks, (B) 8 weeks, (C) 12 weeks and (D) 15 weeks od age. All Taqman qPCR values were normalized to the geometric mean of three housekeeping genes: Actb, Cyc1 and Gapdh. Error bars are SEM (n = 6). Student t-test: *p<0.05, **p<0.01; ***p<0.001. (TIF) [file pone.0108961.s002.tif]
