# Supplementary material for: The Huntington's Disease-Related Cardiomyopathy Prevents a Hypertrophic Response in the R6/2 Mouse Model
Source: PLoS One. 2014 Sep 30;9(9):e108961. doi: 10.1371/journal.pone.0108961 (PMC4182603; doi:10.1371/journal.pone.0108961)
Supplement: Table S1 — Summary of the number of mice per genotype used in all studies and their CAG repeat sizes. SD = standard deviation. (DOC) [file pone.0108961.s003.doc]

**Table S1.**

For the chronic isoproterenol treatment:

| **Treatment group** | **Wild type** | **R6/2** | **CAG (± SD)** |
| --- | --- | --- | --- |
| vehicle | 8 (2 male + 6 female) | 14 (8 male + 6 female) | 213 (**±**2.7) |
| 220µg/g/day | 9 (5 male + 4 female) | 14 (7 male + 7 female) | 213 (**±**2.9) |

For the *Hdac* expression profile:

| **Wild type** | **R6/2** | **CAG (± SD)** |
| --- | --- | --- |
| 24 (12 male + 12 female) | 24 (12 male +12 female) | 210 (**±**6.2) |
